# *Acinetobacter baumannii* Survival under Infection-Associated Stresses Depends on the Expression of Resistance–Nodulation–Division and Major Facilitator Superfamily Efflux Pumps

**DOI:** 10.3390/antibiotics13010007

**Published:** 2023-12-20

**Authors:** Inga V. Leus, Marcela Olvera, Justyna W. Adamiak, Lauren L. Nguyen, Helen I. Zgurskaya

**Affiliations:** Department of Chemistry and Biochemistry, University of Oklahoma, Norman, OK 73072, USA; inga.leus-1@ou.edu (I.V.L.); molvera@ou.edu (M.O.); justyna.adamiak@ou.edu (J.W.A.); lauren.nguyen@ucsf.edu (L.L.N.)

**Keywords:** *Acinetobacter baumannii*, multidrug efflux pumps, acidic stress

## Abstract

Multidrug efflux transporters are major contributors to the antibiotic resistance of *Acinetobacter baumannii* in clinical settings. Previous studies showed that these transporters are tightly integrated into the physiology of *A. baumannii* and have diverse functions. However, for many of the efflux pumps, such functions remain poorly defined. In this study, we characterized two putative drug efflux pumps, AmfAB and AmfCD (Acinetobacter Major Facilitator), that are homologous to EmrAB-like transporters from *Escherichia coli* and other Gram-negative bacteria. These pumps comprise the Major Facilitator Superfamily (MFS) transporters AmfB and AmfD and the periplasmic membrane fusion proteins AmfA and AmfC, respectively. We inactivated and overproduced these pumps in the wild-type ATCC 17978 strain and its derivative strains lacking the major efflux pumps from the Resistance–Nodulation–Division (RND) superfamily and characterized antibiotic susceptibilities and growth of the strains under stresses typical during human infections. We found that neither AmfAB nor AmfCD contribute to the antibiotic non-susceptibility phenotypes of *A. baumannii.* The two pumps, however, are critical for the adaptation and growth of the bacterium under acidic stress, whereas AmfCD also contributes to growth under conditions of low iron, high temperature, and in the presence of bile salts. These functions are dependent on the presence of the RND pumps, the inactivation of which further diminishes *A. baumannii* survival and growth. Our results suggest that MFS transporters contribute to stress survival by affecting the permeability properties of the *A. baumannii* cell envelope.

## 1. Introduction

*Acinetobacter baumannii* is a Gram-negative ESKAPE pathogen that can cause a broad range of severe infections in humans, including hospital-acquired and ventilator-associated pneumonia, urinary tract infections, meningitis, bacteremia, and gastrointestinal, skin, and wound infections [1,2]. This microorganism is well known for its environmental persistence—its ability to grow across a range of temperatures, osmotic conditions, and pHs [3]. Extensive antibiotic use in the hospital environment has allowed this pathogen to develop resistance to all classes of antibiotics and become better suited to out-competing other bacteria that are drug-susceptible [4]. As a result, multidrug-resistant *A. baumannii* infections commonly occur in hospital settings and can spread quickly among patients within those environments [5]. These adaptations make *A. baumannii* a worrisome pathogen, especially for vulnerable and critically ill patients. During the COVID-19 pandemic, from 2019 to 2020, the United States saw a 78% increase in infections starting during hospitalization caused by the particularly concerning carbapenem-resistant *Acinetobacter* spp. [6].

*A. baumannii* utilizes a variety of antibiotic resistance mechanisms, among which the active efflux of antibiotics and biocides is the major contributor in clinical settings [7]. Active efflux affects a broad range of structurally unrelated antibiotics and is the most effective when it works synergistically with other resistance mechanisms such as the low-permeability barrier of the outer membrane and/or antibiotic class-specific resistance mechanisms, e.g., drug inactivation [8]. Both the slow permeation across the outer membrane and antibiotic-inactivating enzymes keeps the effective concentrations of antibiotics below levels needed for the saturation of efflux pumps. As a result, efflux pumps remain highly effective at a wide range of external drug concentrations.

As in other Gram-negative bacteria, the efflux pumps of *A. baumannii* belong to several families of transporters. The families relevant to this work include Resistance–Nodulation–Division (RND) and Major Facilitator Superfamily (MFS); all are secondary transporters driven by a proton-motive force. The most clinically important are transporters from the RND superfamily of proteins [2,9,10]. They are comprised of three components: a periplasmic membrane fusion protein (MFP), an inner membrane transporter protein (IMP), and an outer membrane protein (OMP). The MFP connects the two transmembrane proteins to form a tripartite complex and is integral to the pump’s conformational rotation mechanism that drives the drug transport cycle. The IMP is the transporter itself and the site of proton translocation and is responsible for drug recognition and binding. The OMP is an outer membrane channel, which enables drug extrusion into the extracellular medium [11]. There are at least three RND pumps in *A. baumannii* that can contribute to antibiotic resistance: AdeABC, AdeIJK, and AdeFGH, with the first two frequently found to be overproduced in multidrug-resistant clinical isolates [12]. In addition to drug efflux, both AdeIJK and AdeABC are implicated in different physiological functions. AdeIJK is critical for membrane composition stability [13,14] and vital for lipid homeostasis maintenance, specifically as a lipid export mechanism [15]. Changes in membrane composition that reduce membrane stability in turn affect how well antibiotics are able to cross the outer membrane. Additionally, the export of lipids may function as a mechanism that promotes surface migration [13], perhaps as part of *A. baumannii*’s colonization and pathogenic processes [16]. The overproduction of AdeABC was reported to be associated with fitness diminution in vitro and in vivo in a model that mimics sepsis [13]. In contrast, in a pulmonary infection model, this AdeABC overproducer was more virulent.

We previously found that the inactivation of major RND efflux pumps AdeABC and AdeIJK in *A. baumannii* AB5075 strain leads to changes in antibiotic susceptibilities and to pump-specific changes in transcriptomes and growth phenotypes [12,14]. Among others, the putative efflux pumps from the MFS superfamily ABUW_1930/ABUW_1931 and ABUW_1949/ABUW_1950 that are homologous to EmrAB pump from *E. coli* were significantly overproduced in RND-deficient cells [12]. Based on the previous study’s results, we anticipated that, similar to RND pumps, these putative EmrAB-like pumps would have substrate specificities and some involvement in *A. baumannii*’s antimicrobial non-susceptibility, perhaps as alternative exporters to make up for the loss of RND efflux. Like RND efflux pumps, these transporters function as tripartite complexes. The first genes in the ABUW_1930/ABUW_1931 and ABUW_1949/ABUW_1950 operons encode the periplasmic membrane fusion proteins followed by the MFS transporters. However, genes encoding the OMP components are not present in the operons and are likely encoded somewhere else on the chromosome. EmrAB is known to confer resistance to hydrophobic compounds like nalidixic acid and thiolactomycin in *E. coli* [17], but the substrate specificities of MFS efflux pumps and their potential functions in the drug resistance and physiology of *A. baumannii* remain unknown.

In this study, we generated deletions of genes encoding the putative EmrAB-like multidrug efflux pumps of *A. baumannii* and constructed strains overproducing these transporters in the cells with the wild-type repertoire of RND efflux pumps and cells lacking these transporters. The constructed mutants were characterized for their susceptibilities to representative antibiotics and their abilities to grow under stress conditions associated with human infections. We found that EmrAB homologs have non-complementary functions, but both contribute to the survival of *A. baumannii* under acidic stress.

## 2. Results

### 2.1. MFS Pumps Do Not Contribute to Antibiotic Efflux

The genome of ATCC17978 (AbWT) cells encodes A1S_1772/1773 and A1S_1799/1800 operons corresponding to the ABUW_1949/1950 (98/99% identity) and ABUW_1930/1931 (99/99% identity) operons in AB5075, respectively. We renamed A1S_1772/1773 and A1S_1799/1800 as AmfAB and AmfCD (Acinetobacter Major Facilitator), respectively. To determine the role of these EmrAB-like pumps in *A. baumannii* physiology, we deleted genes encoding these pumps one by one and together in the AbWT and Δ3 (Δ*adeIJK* Δ*adeAB* Δ*adeFGH*) genetic backgrounds (Table S1). We then characterized the susceptibility to antibiotics of the constructed strains.

As reported before, the MIC values of antibiotics belonging to different structural classes of compounds and a detergent SDS are significantly higher in AbWT cells than in their RND-deficient derivative Δ3 (Table 1). Neither the inactivation of AmfAB, AmfCD nor both pumps in AbWT and Δ3 cells affected the MIC values of antibiotics (Table 1). These results show that AmfAB and AmfCD do not contribute to intrinsic levels of antibiotic susceptibility.

To complement the constructed deletion strains, *amfAB* and *amfCD* genes were cloned into the pTJ1 vector enabling the expression of the plasmid-borne genes under an arabinose-inducible promoter as well as the integration of the expression cassette into the Tn7 site on the chromosome. After transformation into the AbWT strain and its derivatives, the corresponding proteins tagged with six His residues at the C-termini were produced from both the plasmid and the Tn7 chromosomal site. Based on immunoblotting with anti-His tag antibodies, both proteins were expressed, albeit to different levels (Figure S1). The expression of AmfC was the strongest, whereas the amounts of AmfB were very low.

The overproduction of Amf pumps in different genetic backgrounds did not increase the MICs of antibiotics (Table 1). Thus, in agreement with the gene deletion analyses, neither of the pumps contribute to the efflux of antibiotics. Furthermore, the AbWT cells carrying either AmfAB or AmfCD plasmids became 2–4-fold more susceptible to erythromycin and zeocin (Table 1), suggesting that the overproduction of these pumps compromises the intrinsic protection of *A. baumannii* cells against these antibiotics.

### 2.2. Both AmfAB and AmfCD Pumps Are Important for Growth under Acidic Conditions, but AmfCD also Contributes to Survival under Other Stresses

**Figure 1:**
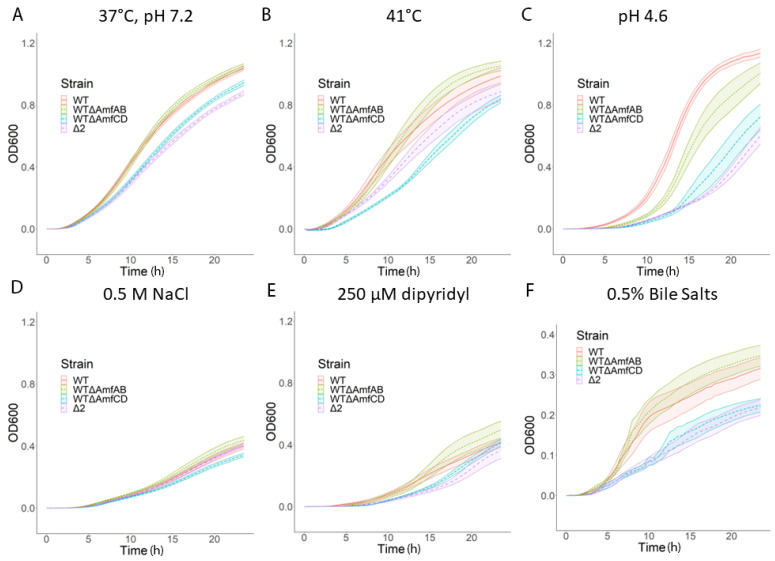
Growth curves of AmfAB and AmfCD deletion strains under indicated conditions. Error bars are SD (*n* = 3). (**A**) No stress control; (**B**) high temperature; (**C**) acidic stress; (**D**) osmotic stress; (**E**) low iron; (**F**) bile stress.

We next compared the growth of AbWT and the constructed deletion strains under the typical laboratory conditions of 37 °C and pH 7.2 and the virulence-induced stresses such as growth at a high temperature of 41 °C, at acidic pH 4.6, in the presence of 0.5 M NaCl (osmotic shock), 125 μM, and 250 μM 2,2′-dipyridyl (a ferrous iron chelator generating low iron stress) and 0.5% bile salts (Table 2). These conditions were used previously to mimic in vitro the stress conditions that are experienced by various pathogens during infections [18]. To compare bacterial growth under different conditions, we calculated the area under the curve for each growth curve collected during a 24 h period (Table 3). We found that AbWT cells grow equally well at 37 °C and 41 °C (Table 3 and Figure 1). At acidic pH, there is a notable lag, but once cells resume growth, it is even faster than that at pH 7.2. All other conditions reduce the growth of AbWT by 3–4-fold.

The inactivation of AmfAB and AmfCD in AbWT led to changes in growth physiology but with transporter-specific results. Under typical laboratory conditions, strains carrying deletions of AmfCD alone (∆AmfCD) and together with AmfAB (∆2) grew somewhat slower than the AbWT, whereas ∆AmfAB cells were indistinguishable from AbWT (Figure 1A and Table 3). The growth rates of ∆AmfCD and ∆2 cells were further reduced when stressed by the presence of bile salts at high temperature and under osmotic shock (Figure 1B–E). In contrast, ∆AmfAB grew either at the same rate as AbWT or even faster. The only exception was acidic stress. At pH 4.6, the growth of all three Amf deletion strains was delayed as seen from extended lag phases, with ∆2 cells recovering the slowest (Figure 1C).

Thus, both AmfAB and AmfCD transporters contribute to the adaptation and survival of *A. baumannii* at pH 4.6, whereas AmfCD also plays a role when cells are stressed by high osmolarity, bile salts and heat.

We next characterized the stress phenotypes of the complemented cells and calculated the corresponding AUCs values (Table 4). For these experiments, all cell cultures were grown in the presence of 1% L-arabinose needed to induce the expression of both plasmid-borne and chromosomally integrated efflux pump genes. Since 250 μM 2,2′-dipyridyl was notably toxic to complemented strains, the iron depletion experiments were carried out at 125 μM 2,2′-dipyridyl.

**Figure 2:**
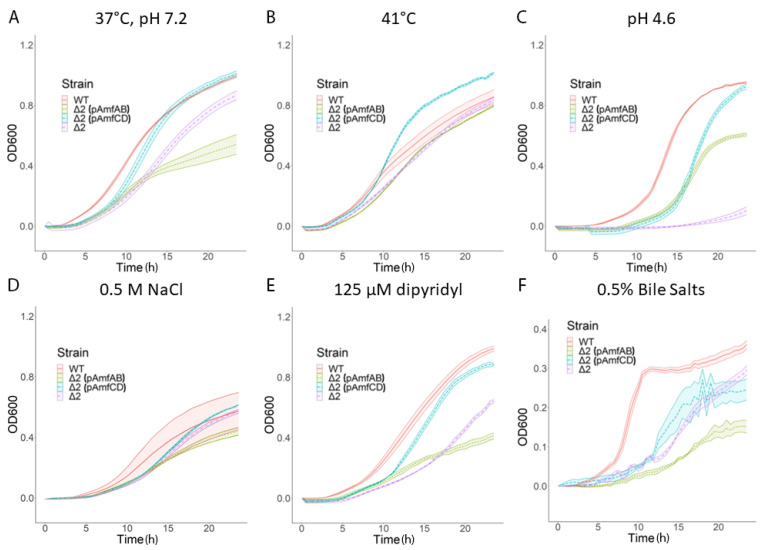
Growth curves of AmfAB and AmfCD overexpression strains under different stresses. Error bars are SD (*n* = 3). (**A**) No stress control; (**B**) high temperature; (**C**) acidic stress; (**D**) osmotic stress; (**E**) low iron; (**F**) bile stress.

In the AbWT and ∆2 cells, the overproduction of AmfAB but not AmfCD was toxic to the cells, as seen from the significant growth reduction in AbWT(pAmfAB) and ∆2(pAmfAB), even under the normal conditions (Table 4 and Figure S2). The toxicity of AmfAB overproduction in the AbWT can also be seen when cells are grown in the pH 4.6 medium (Figure S2). In contrast, the overproduction of either AmfAB or AmfCD rescued the growth deficiency of ∆2 cells grown in pH 4.6 (Figure 2). Thus, in agreement with the inactivation results, both AmfAB and AmfCD contribute to the growth of *A. baumannii* under acidic conditions.

### 2.3. RND Pumps Dominate the Growth Phenotypes of A. baumannii under Virulence-Related Stress Conditions

The antibiotic susceptibility of *A. baumannii* is defined by the activities of RND pumps and their expression is also affected by virulence-related stresses [19]. We next compared the growth of AbWT and its RND-deficient derivative ∆3 under normal and stress conditions.

The inactivation of RND pumps in ∆3 cells did not lead to significant changes in growth under typical laboratory conditions, but these cells grew somewhat slower at 41 °C and under the osmotic stress (Table 3 and Figure S3). Surprisingly, the growth of Ab∆3 cells was strongly impaired at pH 4.6, in the presence of 250 μM 2,2′-dipyridyl and bile salts (Figure S3). Thus, RND pumps are essential for the survival of *A. baumannii* under these conditions.

We next analyzed the growth phenotypes of ∆3 derivatives lacking AmfAB, AmfCD or both transporters. In agreement with the dominant role of the RND transporters, we found no significant changes in the antibiotic susceptibilities of the constructed derivatives of ∆3 (Table 1). Similarly, the inactivation of either AmfAB (∆3∆AmfAB), AmfCD (∆3∆AmfCD) or both (∆5) did not deepen the growth deficiencies of ∆3. Furthermore, ∆3∆AmfCD cells grew somewhat better in acidic and low iron media compared to ∆3. However, even weak growth of ∆3 in bile salts was completely inhibited in mutants lacking AmfAB and AmfCD, suggesting a further weakening of the cell protection against these detergents (Figure S4).

Neither AmfAB nor AmfCD overproduction was able to complement the growth deficiencies of ∆3 and ∆5 cells and their derivatives. Hence, the activities of AmfAB and AmfCD depend on the presence of functional RND pumps. Furthermore, at pH 4.6, the overproduction of either AmfAB or AmfCD was toxic to ∆3 and ∆5 cells and led to the lack of growth (Figures S5 and S6). In contrast, the toxicity of AmfAB overproduction seen in AbWT and ∆2 cells under standard conditions, pH 4.6, high temperature and in the presence of 2,2′-dipyridyl and bile salts (Figure 2 and Figure S2) was also alleviated in ∆3 cells lacking RND pumps (Figure S5). This result suggests that the overproduction of AmfAB in AbWT cells might interfere with the functions of RND pumps.

The results show that strains lacking efflux pumps were most sensitive to the bile salt and 2,2′-dipyridyl stresses. Bile salts are known to be substrates of RND efflux pumps, and the growth impairment could be due to the lack of efflux of these detergents and their effect on the integrity of membranes [20,21]. Although 2,2′-dipyridyl is broadly used to study the effect of iron limitation on bacterial physiology [22,23,24], it could be a substrate of RND and other efflux pumps, and the inhibition of the growth of the deletion strains could be due to the intracellular accumulation of this compound and not due to iron deficiency. Therefore, we also tested whether ∆3 and other deletion mutants could grow in the M9 citrate minimal medium with and without a ferric iron supplement (Figure S7). We found that all efflux deletion strains grew similarly in the minimal medium in the presence and absence of FeCl_3_, but slower under the iron-deficient conditions. Thus, 2.2′-dipyridyl itself contributes to growth deficiencies of the Ade and Amf knockout strains.

### 2.4. Principal Component Analysis Reveals Differences between the Strains

**Figure 3:**
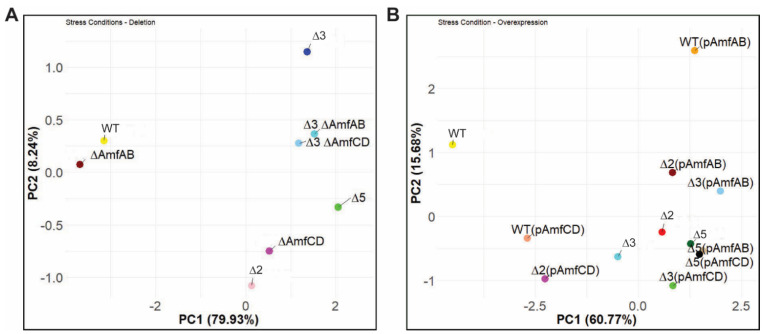
PCA analyses of *A. baumannii* growth. The areas under curves (AUCs) were calculated for the deletion mutants (**A**) and the complemented strains (**B**) and analyzed using hierarchical clustering. The principal component decomposition in the first and second principal components (PC1 and PC2) coordinates is shown.

To compare the growth phenotypes of the constructed strains, we carried out the principal component analysis (PCA) of the AUC values calculated for the averaged growth curves of all strains under the analyzed growth conditions (Table 3 and Table 4). As seen in Figure 3A, the first two principal components (PCs) explain 88.2% of the total variance in the dataset. The PC1 separates the AbWT and ∆AmfAB strains from the rest of the strains. These two strains grew well under all tested conditions. In contrast, all other deletion derivatives are very distant from AbWT and ∆AmfAB and are separated by PC2. The ∆AmfCD and ∆2 phenotypes are grouped together demonstrating the dominance of *amfCD* deletion in the phenotypes of ∆2 cells. The phenotypes of ∆3 cells are clearly distinct from ∆5, with ∆3∆AmfAB and ∆3∆AmfCD located in between ∆3 and ∆5. Thus, despite the strong effect of RND pumps inactivation on growth under stresses, AmfAB and AmfCD remain functional and contribute to the observed growth phenotypes.

The relationships between overproducers are more complex, reflecting the dependence of the phenotypes on specific plasmid-borne pumps and genetic backgrounds (Figure 3B). The PC1 and PC2 explain ~76% of the total variance and separated AbWT from the rest of the strains, demonstrating that overproduction of either AmfAB or AmfCD does not fully complement their deletion phenotypes. In addition, WT(pAmfAB) and WT(pAmfCD) are distant from each other and other strains. This separation is likely due to the finding that the overproduction of AmfAB was the most toxic to the cells.

### 2.5. Efflux Pump Deletions and Overproducers Modify the Permeability Barrier of A. baumannii

The involvement of efflux pumps in acid tolerance suggests that their functions are important for the adaptation to this stress. Previous studies implicated the RND pumps of *A. baumannii* in the maintenance of lipid composition of the cellular membranes [14,15]. Since membrane modification is one of the established mechanisms of acid tolerance in bacteria, we next analyzed whether the deletions and plasmid-borne overproductions of Amf pumps affect the permeability barrier of *A. baumannii.* For this purpose, we analyzed the intracellular accumulation of a membrane fluorescent probe N-phenyl-1-naphthylamide (NPN). NPN is a hydrophobic probe, which penetrates slowly across the outer membrane and is a known substrate of AdeIJK pump [25]. Therefore, the fluorescence of NPN is dramatically enhanced when AdeIJK is inactivated and/or when the outer membrane is permeabilized.

**Figure 4:**
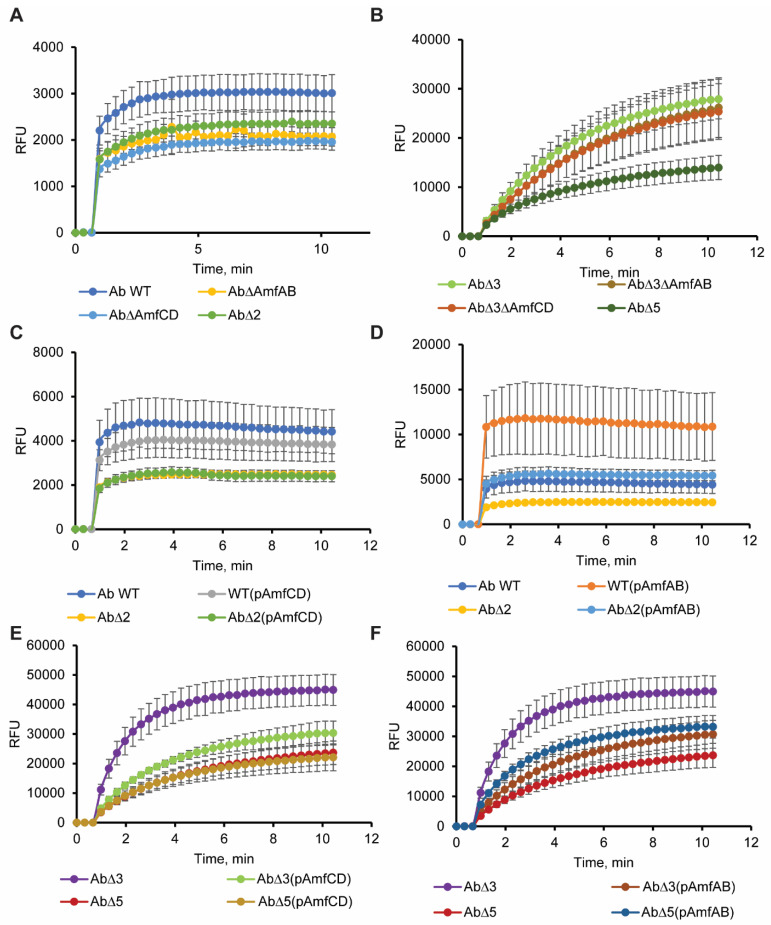
Intracellular accumulation of the fluorescent membrane probe NPN. Indicated strains were grown in LB broth and induced with 1% L-arabinose when appropriate. Washed cells were injected into HMG buffer containing 4 μM of NPN and the fluorescence was monitored continuously for 10 min. The plots are changes in fluorescence intensity as a function of time. Error bars are SD (*n* = 3). (**A**) Single and double deletions of AmfAB and AmfCD in the Ab WT genetic background. (**B**) Single and double AmfAB and AmfCD deletions in Ab∆3 genetic background. (**C**) Overproduction of AmfCD in Ab WT and Ab∆2 backgrounds. (**D**) Overproduction of AmfAB in Ab WT and Ab∆2 backgrounds. (**E**) Overproduction of AmfCD in Ab∆3 and Ab∆5 backgrounds. (**F**) Overproduction of AmfAB in Ab∆3 and Ab∆5 backgrounds.

Neither inactivation of AmfAB nor AmfCD increased the intracellular accumulation of NPN in AbWT cells, suggesting that the permeability barriers of these cells are not compromised and even if NPN is the substrate of these pumps they are not the major contributors to its efflux in AbWT (Figure 4A). Furthermore, all three deletion mutants ∆AmfAB, ∆AmfCD and the double knockout ∆2 were less fluorescent than AbWT. Hence, the inactivation of these pumps makes *A. baumannii* cells less permeable to NPN. As expected, ∆3 cells lacking the RND pumps accumulated at least ten times higher amounts of NPN than AbWT (Figure 4B). Although, single knockouts ∆3∆AmfAB and ∆3∆AmfCD were indistinguishable from the parental ∆3 cells, in the double knockout ∆5 cells the levels of NPN fluorescence were at least three times lower. We conclude that deletions of AmfAB and AmfCD pumps either promote the efflux of NPN from cells by other transporters or reduce the permeation of NPN across the outer membrane in both RND plus and minus backgrounds.

The effect of overproduction on the permeability barrier was both the pump- and genetic background-specific (Figure 4C–F). The increased fluorescence of NPN in AbWT(pAmfAB), ∆2(pAmfAB) and ∆5(pAmfAB) suggests that these cells are leaky to NPN and this result is consistent with the toxicity of the overproduction of AmfAB pump seen in the growth curves (Table 4). In contrast, AmfCD overproduction has no effect on the NPN accumulation and on the growth of AbWT(pAmfCD), ∆2(pAmfCD) and ∆5(pAmfCD) cells. Surprisingly, the overproduction of either of the two pumps in ∆3 cells lead to decreased NPN accumulation and, hence, a stronger permeability barrier, highlighting the physiological difference between ∆3 and ∆5 cells in how they respond to the overproduction of the Amf pumps.

## 3. Discussion

All bacterial genomes encode multiple efflux transporters, but the functions of many of these transporters are unknown or undefined. The commonly used approach to the characterization of such transporters is to delete the genes and/or overproduce them in RND-deficient genetic backgrounds and to analyze their possible contribution to drug efflux [26,27]. In this study, we applied this approach to *A. baumannii* transporters AmfAB and AmfCD, the abundances of which increased in cells lacking the two major multidrug efflux pumps AdeIJK and AdeABC [12]. We found that neither deletion nor overproduction of AmfAB and AmfCD in the RND plus and minus genetic backgrounds resulted in pump-specific decreases in antibiotic susceptibilities (Table 1). Thus, in *A. baumannii,* these pumps do not contribute to antibiotic non-susceptibility. In contrast, AbWT(pAmfAB) cells became more susceptible to erythromycin and zeocin, the result consistent with the finding that the overproduction of AmfAB is toxic to AbWT and make the cells leaky to NPN (Figure 4D). The lack of contribution to antibiotic non-susceptibility is unexpected because the EmrAB-TolC pump from *E. coli*, the best characterized homolog of Amf pumps, was the first multidrug efflux pump identified in bacteria [17] and is able to expel select antibiotics, dyes and detergents from cells [27].

Unlike the susceptibility to antibiotics, bacterial growth and survival under various stresses depend on Amf pumps. Both AmfAB and AmfCD contribute to *A. baumannii* growth in the pH 4.6 medium, with AmfCD playing a larger role (Figure 1 and Table 3). In addition, RND pumps are also important for the survival under this stress as seen from the large delay in the growth of ∆3 cells in pH 4.6 (Figure S3). Efflux appears to be a common adaptation to acidic stress among various bacterial species [28,29]. In *E. coli,* the inactivation of the RND pump MdtEF-TolC reduces the bacterial fitness in acidic pH and survival in macrophages [30]. Similarly, the MFS pump EmrKY-TolC of *Shigella flexneri* was reported to be important for survival in macrophages and is activated in response to acidic pH [31]. However, it remains unclear how efflux pumps contribute to acid adaptation.

One possibility is that efflux pumps play a role in the consumption of protons needed to sustain pH homeostasis in the cytoplasm. The production of ammonia by *A. baumannii* has been reported to be important for the survival and replication in macrophages [32]. However, ammonia and CO_2_ released during amino acid transformations can rapidly diffuse across membranes, whereas uptake and efflux of amino acids are carried out by specific amino acid exchangers such as ArcD for arginine/ornithine or CadB for lysine/cadaverine [29].

Alternatively, efflux pumps could contribute to modifications of cellular membranes needed for survival in acidic conditions. We previously found that either inactivation or overproduction of Ade pumps change dramatically the lipidome of *A. baumannii* ATCC17978 cells [14]. In addition, the AdeIJK pump was also implicated in the efflux of host fatty acids [15]. Thus, the role of RND and possibly Amf pumps could be in the modification of the fatty acid composition of the membranes during acidic stresses. Our NPN accumulation analyses are consistent with this conclusion and showed that inactivation of either AmfAB or AmfCD makes cells less permeable to this membrane probe (Figure 4). These changes in the permeability barrier were independent from the presence of RND pumps but the effect was the strongest when both Amf pumps were inactivated in ∆5 cells (Figure 4B). This result is consistent with the finding that Amf pumps contribute to survival at pH4.6 and that their inactivation has an additive effect (Table 3).

On the other hand, overproduction of these Amf pumps appears to impair *A. baumannii* growth under acidic conditions (Figure 2), perhaps because proton antiport leads to an influx of protons, making the cell interior even more acidic. Both AmfB and AmfD pumps share amino acid sequence similarity to MdfA efflux transporter, at 26.85% and 31.03%, respectively. Besides conferring antibiotic resistance when expressed in *E. coli*, MdfA also functions as a Na+ (K+)/H+ antiporter which helps maintain intracellular pH homeostasis [33]. It is also possible that the functions of these pumps are similar to those of RosA/RosB efflux pumps of *Yersinia*, in that they mediate a stress response (antibiotic or other) by pumping out said stressor and by inducing the acidification of the cytoplasm. The lower intracellular pH acts as a positive regulatory signal in *Yersinia* that then triggers other cellular stress response systems, perhaps involving modifications to the outer membrane [34,35].

Interestingly, the two pumps AmfAB and AmfCD have non-overlapping physiological functions. The ∆AmfCD cells but not ∆AmfAB grow slower than AbWT under all tested conditions (Figure 1), whereas the overproduction of AmfAB but not AmfCD is toxic to the cells (Figures S2, S5 and S6). The differences between the two pumps are also seen in the published RNAseq data [18]. Under normal conditions, the abundance of *amfC* transcripts is 4–5 times higher than that of *amfA* and further increases at pH 4.6, but decreases upon exposure to bile salts and low-iron conditions (Figure S8). In contrast, *amfA* transcription is induced by bile salts and low-iron conditions. The AmfAB- and AmfCD-associated phenotypes were negated in the RND minus background, suggesting that the activity and/or components of the RND pumps are needed for Amf phenotypes. Since neither of these operons contains a gene encoding the outer membrane channel, it is possible that they engage AdeK for their activities and thus reduce the activity of AdeIJK. The exception is the bile stress, where a loss of either AmfAB or AmfCD was enough to completely inhibit growth (Figure S4). The transport of bile molecules by these Amf pumps possibly reduces the effect of additional stress from the destabilization of membranes caused by these detergents [36].

It is already known that bacterial responses to different stress conditions often overlap. For example, the exposure to high osmolarity and acidic pH triggers the activation of the PhoPQ two-component response system as well as Fur activator regulating the expression of iron acquisition systems [37,38,39]. Perhaps all observed changes in growth due to deletions and overexpression of efflux pumps are caused by the same modifications or the lack of them in the composition of bacterial membranes.

Our results suggest that the deletion of these Amf pumps under various stresses hinders the cells’ capacity to maintain intracellular pH homeostasis and to export stress-associated small toxic molecules. This is in contrast to the membrane disruption seen in the overproduction of the Amf pumps. Overproduction also represents an inefficient management of cellular resources that could be used by stress response systems such as cell envelope remodeling that reduces permeability to reduce further stress damage [40,41]. Future studies of regulatory circuits in *A. baumannii* controlling the expression of efflux pumps could lead to the identification of underlying mechanisms.

These results highlight complex relationships between antibiotic non-susceptibility and *A. baumannii* metabolism [40]. Metabolomics studies could provide the insights needed for understanding the Amf pump–stress mechanisms.

## 4. Materials and Methods

### 4.1. Strains and Growth Conditions

The strains and plasmids used in this study are listed in Table S1. Luria–Bertani (LB) broth (10 g of Bacto tryptone, 5 g of yeast extract, and 5 g of NaCl per liter, pH 7.0) or LB agar (LB broth supplemented with 15 g/L of agar) were used for bacterial growth. When indicated, cultures were induced with 1% L-arabinose (ARA) to induce the overexpression of efflux pumps. All chemicals used were of molecular biology grade. For the selection of *A. baumannii* mutant strains, gentamicin (50 µg/mL), trimethoprim (100 µg/mL), carbenicillin (200 µg/mL) and streptomycin (100 µg/mL) were used.

### 4.2. Deletion of A1S_1772–1773, A1S_1799–1800 Operons

A suicide vector harboring a kanamycin resistance marker pMo130 was first modified by inserting a gentamicin resistance cassette using SphI and BamHI restriction sites to construct pIL105. DNA fragments (approximately 0.5 kb) located upstream and downstream from the respective operons were amplified by PCR using the genomic DNA of *A. baumannii* ATCC 17978 extracted with GenElute™ Bacterial Genomic DNA Kit (Sigma-Aldrich, Darmstadt, Germany) as a template. The PCR fragments were cloned into pIL105 to create deletion cassettes with a gentamicin resistance marker flanked by the PCR fragments upstream (NsiI/SphI) and downstream (BamHI/NotI) from the indicated operons. The constructed pIL147 and pIL148 plasmids were used as templates to amplify ∆AmfAB::Gm and ∆AmfCD::Gm fragments, respectively. All cloning primers can be found in Table S2.

The inactivation of genes was carried out from AbWT and Ab∆3 using RecAB as described by Tucker et al., 2014 [42]. We used electrocompetent cells at a density of 10^10^ CFU/reaction and 5 µg of PCR products with 0.5 kb of flanking homology; we obtained approximately 10–50 colonies from each transformation, depending on the gene being targeted for replacement.

The gentamicin resistance cassette was removed by transformation of the pAT03 plasmid and activation of the FLP recombinase enzyme [42]. Genomic deletions in the mutants were verified by comparing the PCR amplimers obtained from the wild-type strain and corresponding pump gene deletion mutants using external (EXT) primers (Table S2).

### 4.3. Construction of Plasmids for Overproduction of Efflux Pumps

To amplify genes encoding AmfAB and AmfCD efflux pumps, two sets of primers were designed with NcoI/EcoRI and NotI restriction sites for cloning (Table S2). The DNA fragments were amplified by PCR using the genomic DNA of *A. baumannii* AB5075 extracted with GenElute™ Bacterial Genomic DNA Kit (Sigma, St. Louis, MO, USA) as a template. We previously found that the cloning vector pTJ1 replicates in *A. baumannii* ATCC 17978 and can be used for the significant overexpression of genes from both the chromosome and the plasmid [25]. The corresponding PCR products were cloned into pTJ1 to generate pMOM101 and pMOM102 plasmids expressing the efflux pump under P_BAD_ promoter for integration into the Tn7 site. After transformation, clones containing plasmids were selected on 100 µg/µL Tmp plates and successful insertions were confirmed by restriction analyses.

Triparental mating was used to construct AbWT, Ab∆2, Ab∆3 and Ab∆5 cells overproducing the individual efflux pumps. For this purpose, plasmids were transformed into SM10 cells and mated with SM10(pTNS3) and Ab cells as described before [25]. The chromosomal insertions were confirmed by PCR using Ab (17978) *glmS* FWD, Ab (17978) *glmS* REV and PTn7R primers (Table S2).

### 4.4. Drug Susceptibility Assay (Minimal Inhibitory Concentration (MIC) Determination)

Susceptibility to different classes of antibiotics was determined by the two-fold broth dilution method as described before [43]. Briefly, the cells were grown in LB broth with appropriate antibiotics wherever necessary at 37 °C with shaking at 200 rpm. MICs of different classes of antimicrobial agents (Table 1) were measured in 96-well micro-titer plates. For this purpose, exponentially growing cells were inoculated at a density of 10^5^ cells per mL into wells containing just LB or LB medium supplemented with 1% ARA in the presence of two-fold-increasing concentrations of drugs under investigation (ten concentrations in total). The first concentration in the experiment was drug-specific and is usually 2–4 fold higher than the MIC of Ab WT, if it is possible to determine. There were two control wells per plate with no antibiotics +/− cells for quality control. Cell growth was determined visually after incubation of the micro-titer plates at 37 °C for 16–20 h. All measurements were performed in duplicates and repeated at least two times independently.

### 4.5. NPN Uptake

These experiments were carried out as described before [25]. Briefly, overnight cells were subcultured and grown for 1 h in fresh LB medium, then induced, if needed, and grown for four more hours. Cells were collected by centrifugation, washed, and resuspended in 50 mM HEPES-KOH buffer (pH 7.0) containing 1 mM MgSO_4_ and 0.4 mM glucose (HMG buffer) to an OD_600_ of ~1.0. The uptake assay was performed in a temperature-controlled multimode microplate reader (Tecan Spark 10M, Männedorf, Switzerland) equipped with a sample injector in fluorescence mode. The fluorescence of NPN was monitored at λex = 350 nm and λem = 405 nm at a gain of 75 for 10 min. All measurements were collected in duplicates and performed with at least two biological and two technical repeats.

### 4.6. Bacterial Growth with Stress Exposures

Cells from frozen stocks were inoculated into LB medium containing appropriate antibiotics if needed and incubated for 16 h at 37 °C. Exponentially growing cells were inoculated at a density of 10^5^ cells per mL into wells containing LB or LB + 1%ARA medium with and without stress condition. The strength of the stress with the different agents was designed to be as similar and relevant as possible for *A. baumannii* (Table 2) [18]. Bacterial growth assay was performed in a temperature-controlled micro-plate reader (Tecan Spark 10M, Männedorf, Switzerland). The optical density of cells was measured every 30 min in clear 96-well micro-titer plates over 24 h. All measurements were performed in triplicate and repeated at least three times independently. After averaging, area under the curves [44,45] (AUC) were calculated for each strain under different exposures using the formula:

AUC = (OD_n+1_ + OD_n_)/2 × (T_n+1_ − T_n_).

To test the effect of the iron deficiency on the bacterial growth in the absence of 2,2′-dipyridyl, we followed the same protocol, but instead of using LB broth, the deletion strains were grown in the M9 citrate minimal medium [46] with and without a ferric iron supplement (3.1 µM).

### 4.7. Protein Expression and Analyses

For AmfAB and AmfCD protein expression, IL186 and IL187 cells were grown overnight in LB medium supplemented with 100 µg mL^−1^ trimethoprim, and subcultured 1:100 in fresh LB. Cultures were grown at 37 °C with shaking for 1.5 h before induction with 1% arabinose for 4–5 h. Cells were harvested and lysed, and the membrane fraction was solubilized as described before [47]. Immobilized metal affinity chromatography was performed for soluble membrane fractions. Elution fractions in the reducing SDS-sample buffer were analyzed by SDS-PAGE followed by immunoblotting with primary monoclonal anti-6× His antibodies (Sigma), followed by a secondary alkaline phosphatase-conjugated anti-mouse immunoglobulin antibody (Sigma). 5-bromo-4-chloro-3-indolylphosphate (BCIP) and nitroblue tetrazolium (NBT) were used to visualize the bands.

### 4.8. Clustering

Multivariate statistical techniques (principal component analysis, PCA) were applied to investigate the association between strains depending on their physiological properties (growth under stress conditions, NPN uptake, MICs). Data for conducting PCA were normalized by centering to the mean and dividing by the standard deviation. All code, unless indicated, was written in house using RStudio 1.4 and R version 4.04.

### 4.9. Statistical Analysis

All experiments presented in the paper (growth under stress conditions, NPN uptake, MICs) were performed in at least two technical and two independent repeats. For reproducibility, standard statistical analyses were performed. Data were averaged over all biological repeats, and standard error was calculated if necessary.

## Figures and Tables

**Table 1:** Minimal inhibitory concentrations [µg/mL] of strains lacking or overproducing AmfAB and AmfCD efflux pumps.

Strain	Ery	Novo	Cipro	Tet	Azi	Cm	SDS	Gm	Zeo
AbWT	10	10–20	0.125–0.25	0.25–0.5	0.63–1.25	64	>1024	4–8	8
∆AmfAB	5	10	0.125–0.25	0.5	0.63–1.25	64	>1024	8	8
∆AmfCD	5–10	5–10	0.125	0.25–0.5	1.25	64	>1024	8	8
∆2	10	10	0.125	0.25	1.25	32	≥1024	R	8
AbWT(pAmfAB)	**2.5–5**	5–10	0.125	0.5–1	0.31–0.63	64	>1024	4	2
AbWT(pAmfCD)	**2.5–5**	10–20	0.125	0.5	0.63	64	>1024	4	4
∆2(pAmfAB)	10	5–10	0.0625–0.125	0.5	0.63–1.25	32	>1024	R	4–8
∆2(pAmfCD)	5	5–10	0.0625–0.125	0.25	1.25	32	>1024	R	16
Ab∆3	1.25	0.08	0.016–0.031	0.031–0.063	0.31–0.63	16	16	8	1
∆3∆AmfAB	1.25	0.08	0.008–0.016	0.031	0.63	8–16	16	8	1
∆3∆AmfCD	1.25	0.08	0.016	0.031	0.63	8–16	16	8	1–2
∆5	2.5	0.08	0.016	0.031	0.31–0.63	8	16	R	1–2
∆3(pAmfAB)	**5**	0.04	0.016	0.016–0.031	0.31	8	16	8	1–2
∆3(pAmfCD)	**2.5–5**	0.04	0.016	0.016	0.31–0.63	8	16	8	0.5–1
∆5(pAmfAB)	5	0.04	0.016	0.016–0.031	0.63	8	16	R	0.25–0.5
∆5(pAmfCD)	2.5–5	0.04	0.016	0.016–0.031	0.63	8	16	R	0.5–1

Ery—erythromycin, Novo—novobiocin, Cipro—ciprofloxacin, Tet—tetracycline, Azi—azithromycin, Cm—chloramphenicol, SDS—sodium dodecyl sulfate, Gm—gentamicin, Zeo—zeocin, R—resistant. MIC values that differ by four-fold are shown in underlined bold.

**Table 2:** Details of growth and stress conditions for *A. baumannii* mutants.

Type of Stress	Stress Exposure Agent in LB Broth or LB Broth + 1% L-Arabinose
No stress control	LB broth, pH 7.2; 37 °C
Acidic stress	LB broth adjusted to pH 4.6 with HCl
Osmotic stress	LB broth supplemented with 0.5 M NaCl
Bile stress	LB broth supplemented with 0.5% bile salts (Millipore (Darmstadt, Germany), ~50% sodium cholate and ~50% sodium deoxycholate)
Low iron	LB broth supplemented with 125 μM or 250 μM 2,2-dipyridyl
Temperature	LB broth, pH 7.2; 41 °C

**Table 3:** Area under curve (AUC) values for the growth curves of the parental and deletion strains collected under indicated stress conditions. Standard deviations (SDs, *n* = 3) for each experiment are shown.

Strains	No Stress	Acid	Osmotic	Bile	Iron Depletion	High Temperature
	37 °C, pH 7.2	pH 4.6	0.5 M NaCl	0.5% Bile Salts	2,2′-Dipyridyl (250 mM)	41 °C
WT	12.13 ± 0.96	11.71 ± 2.91	3.48 ± 1	4.16 ± 2.19	3.66 ± 2.46	12.1 ± 4.21
∆AmfAB	12.34 ± 1.17	8.26 ± 2.1	3.89 ± 0.64	4.65 ± 1.47	4.13 ± 2.25	12.92 ± 2.79
∆AmfCD	10.16 ± 0.86	4.59 ± 2.48	2.92 ± 0.26	2.6 ± 0.81	2.78 ± 0.54	7.96 ± 0.94
∆2	9.42 ± 0.92	3.59 ± 0.9	3.42 ± 0.36	2.45 ± 0.77	2.36 ± 1.11	9.98 ± 2.92
∆3	10.81 ± 1.43	1.67 ± 0.81	2.59 ± 0.65	0.46 ± 0.28	1.69 ± 1.38	10.04 ± 3.46
∆3∆AmfAB	10.65 ± 0.67	1.44 ± 0.71	3.14 ± 0.48	NG	1.12 ± 0.45	8.91 ± 3.05
∆3∆AmfCD	9.89 ± 0.87	2.57 ± 1.27	2.97 ± 0.44	NG	1.94 ± 0.59	10.17 ± 2.9
∆5	9.16 ± 0.9	2.06 ± 1.21	2.99 ± 0.51	NG	1.14 ± 0.76	8.98 ± 2.23

NG, no growth. The color indicates where each value falls within the range: red—the highest growth and blue—no growth.

**Table 4:** Area under curve (AUC) values for the growth curves of the parental and deletion strains complemented with plasmids overproducing efflux pumps in the presence of 1% L-arabinose. Standard deviations (SDs, *n* = 3) for each experiment are shown.

Strains	No Stress	Acid	Osmotic	Bile	Iron Depletion	High Temperature
	37 °C, pH 7.2	pH 4.6	0.5 M NaCl	0.5% DOC	2,2′-Dipyridyl (125 mM)	41 °C
WT	11.89 ± 1.47	9.39 ± 0.61	5.94 ± 2.36	4.78 ± 0.58	9.93 ± 5.7	9.78 ± 3.03
WT(pAmfAB)	5.21 ± 1.23	4.85 ± 1.04	1.61 ± 0.12	2.62 ± 0.24	5.41 ± 0.08	5.33 ± 0.57
WT(pAmfCD)	11.83 ± 0.69	7.91 ± 1.9	3.47 ± 0.39	3.77 ± 0.27	8.67 ± 0.13	11.95 ± 0.57
D2	8.19 ± 0.53	0.16 ± 0.21	5.04 ± 0.49	2.47 ± 0.17	4.28 ± 0.17	8.48 ± 0.59
D2(pAmfAB)	6.12 ± 0.7	4.3 ± 0.7	4.33 ± 0.44	1.3 ± 0.32	4 ± 0.67	8.15 ± 0.12
D2(pAmfCD)	10.95 ± 0.7	5.49 ± 0.96	5.43 ± 0.11	2.71 ± 1.1	7.96 ± 0.4	11.88 ± 0.21
D3	10.62 ± 1.27	1.13 ± 0.13	3.19 ± 0.78	NG	7.52 ± 4.82	9.1 ± 2.14
D3(pAmfAB)	7.2 ± 1.78	0.08 ± 0.08	0.98 ± 0.62	0.39 ± 0.7	4.69 ± 0.01	7.58 ± 0.4
D3(pAmfCD)	9.65 ± 1.17	0.04 ± 0.07	2.32 ± 0.44	0.12 ± 0.31	4.96 ± 0.19	9.95 ± 0.91
D5	8.01 ± 1.01	NG	4.81 ± 0.59	0.57 ± 0.42	4.45 ± 0.32	7.61 ± 0.4
D5(pAmfAB)	7.3 ± 1.08	NG	3.96 ± 0.59	0.62 ± 0.6	3.49 ± 0.69	8.73 ± 0.37
D5(pAmfCD)	8.07 ± 0.75	NG	4.65 ± 0.31	0.14 ± 0.35	4.04 ± 0.74	7.73 ± 0.29

NG, no growth. The color indicates where each value falls within the range: red—the highest growth and blue—no growth.

## Data Availability

The data presented in this study are available in Supplementary Materials.

## Supplementary Materials

The following supporting information can be downloaded at: https://www.mdpi.com/article/10.3390/antibiotics13010007/s1, Table S1. *A. baumannii* strains and plasmids used in this study. Table S2. Primers used in this study. Figure S1. Expression of AmfB and AmfC proteins in *A. baumannii* IL186 and IL187 cells. Figure S2. Growth curves of AbWT, ∆2 and AbWT overproducing AmfAB and AmfCD pumps under indicated growth conditions. Figure S3. Growth curves of AbWT and its RND-deficient ∆3 cells under indicated growth conditions. Figure S4. Growth curves of the RND-deficient ∆3 and its derivatives lacking AmfAB, AmfCD or both pumps under indicated growth conditions. Figure S5. Growth curves of ∆3 overproducing AmfAB and AmfCD pumps under indicated growth conditions. Growth of ∆5 cells is shown for comparison. Figure S6. Growth curves of ∆5 overproducing AmfAB and AmfCD pumps under indicated growth conditions. Growth of ∆3 cells is shown for comparison. Figure S7. Growth curves of AbWT, the RND-deficient ∆3 and their derivatives lacking both AmfAB and AmfCD pumps in the M9 based medium supplemented with 0.5% sodium citrate as a sole carbon source and with or without 3.1 μM of FeCl_3_. Figure S8. RNAseq analyses of abundances of *amfA* and *amfC* transcripts. References [48,49,50] are only cited in the Supplemenatry Materials.
