# Supplementary material for: Acinetobacter baumannii Survival under Infection-Associated Stresses Depends on the Expression of Resistance–Nodulation–Division and Major Facilitator Superfamily Efflux Pumps
Source: Antibiotics (Basel). 2023 Dec 20;13(1):7. doi: 10.3390/antibiotics13010007 (PMC10812440; doi:10.3390/antibiotics13010007)
Supplement: Supplementary file 1 [file antibiotics-13-00007-s001.zip › antibiotics-2752634-supplementary.pdf]

Supplementary information

***Acinetobacter baumannii* survival under infection-associated stresses depends on the expression of RND and MFS efflux pumps.**

Inga V. Leus, Marcela Olvera, Justyna W. Adamiak, Lauren Nguyen, Helen I. Zgurskaya

Content:

Table S1. *A. baumannii* strains and plasmids used in this study.

Table S2. Primers used in this study.

Figure S1. Expression of AmfB and AmfC proteins in *A. baumannii* IL186 and IL187 cells.

Figure S2. Growth curves of AbWT, Δ2 and AbWT overproducing AmfAB and AmfCD pumps under indicated growth conditions.

Figure S3. Growth curves of AbWT and its RND-deficient Δ3 cells under indicated growth conditions.

Figure S4. Growth curves of the RND-deficient Δ3 and its derivatives lacking AmfAB, AmfCD or both pumps under indicated growth conditions.

Figure S5. Growth curves of Δ3 overproducing AmfAB and AmfCD pumps under indicated growth conditions. Growth of Δ5 cells is shown for comparison.

Figure S6. Growth curves of Δ5 overproducing AmfAB and AmfCD pumps under indicated growth conditions. Growth of Δ3 cells is shown for comparison.

Figure S7. Growth curves of AbWT, the RND-deficient Δ3 and their derivatives lacking both AmfAB and AmfCD pumps in the M9 based medium supplemented with 0.5% sodium citrate as a sole carbon source and with or without 3.1 μM of $FeCl_3$.

Figure S8. RNAseq analyses of abundances of *amfA* and *amfC* transcripts.

Table S1. ***A. baumannii*** **strains and plasmids used in this study.**

**Strain**	**Relevant genotype**	**Source**
ATCC17978	Drug-susceptible wild type	ATCC
JWW30 (17978 WT)	*A.baumannii* ATCC17978 resistant to streptomycin	[1]
IL188 (WTΔAmfAB)	JWW30 Δ*A1S_1772-73*	This study
IL189 (WTΔAmfCD)	JWW30 Δ*A1S_1799-1800*	This study
IL190 (WTΔ2)	IL189 Δ*A1S_1772-73::Gm*^{r}	This study
IL119 (AbΔ3)	JWW30 Δ*adeIJK* Δ*adeAB* Δ*adeFGH*	[1]
IL198 (Δ3 ΔAmfAB)	IL119 Δ*A1S_1772-73*	This study
IL199 (Δ3 ΔAmfCD)	IL119 Δ*A1S_1799-1800*	This study
IL200 (Δ5)	IL199 Δ*A1S_1772-73::Gm*^{r}	This study
IL186 (WT pAmfAB)	JWW30 *att*Tn*7*::mini-Tn*7*T-Tp^{r}-*araC*-P_{BAD}-*ABUW_1949-50* carrying pMOM101	This study
IL187 (WT pAmfCD)	JWW30 *att*Tn*7*::mini-Tn*7*T-Tp^{r}-*araC*-P_{BAD}- *ABUW_1930-31* carrying pMOM102	This study
MOM101 (Δ2 pAmfAB)	IL190 *att*Tn*7*::mini-Tn*7*T-Tp^{r}-*araC*-P_{BAD}-*ABUW_1949-50* carrying pMOM101	This study
MOM102 (Δ2 pAmfCD)	IL190 *att*Tn*7*::mini-Tn*7*T-Tp^{r}-*araC*-P_{BAD}- *ABUW_1930-31* carrying pMOM102	This study
IL194 (Δ3 pAmfAB)	IL119 *att*Tn*7*::mini-Tn*7*T-Tp^{r}-*araC*-P_{BAD}-*ABUW_1949-50* carrying pMOM101	This study
IL195 (Δ3 pAmfCD)	IL119 *att*Tn*7*::mini-Tn*7*T-Tp^{r}-*araC*-P_{BAD}- *ABUW_1930-31* carrying pMOM102	This study
MOM103 (Δ5 pAmfAB)	IL200 *att*Tn*7*::mini-Tn*7*T-Tp^{r}-*araC*-P_{BAD}-*ABUW_1949-50* carrying pMOM101	This study
MOM104 (Δ5 pAmfCD)	IL200 *att*Tn*7*::mini-Tn*7*T-Tp^{r}-*araC*-P_{BAD}- *ABUW_1930-31* carrying pMOM102	This study
Plasmids		
pTNS3	Amp^{r}; Helper plasmid encoding Tn*7* transposase proteins TnsABCD from P1 and P_{lac} promoter	[2]
pTJ1	pUC18T-mini-Tn*7*T-Tp-*araC*-P_{BAD}-MCS, Amp^{r}, Tp^{r}	[3]
pMOM101 (pTJ1-AmfAB)	pUC18T-mini-Tn*7*T-Tp-*araC*-P_{BAD}-*ABUW_1949-50*, Amp^{r}, Tp^{r}	This study
pMOM102 (pTJ1-AmfCD)	pUC18T-mini-Tn*7*T-Tp-*araC*-P_{BAD}-*ABUW_1930-31*, Amp^{r}, Tp^{r}	This study
pAT02	pMMB67EH with Rec_{Ab} system, Amp^{r}	[4]
pAT03	pMMB67EH with FLP recombinase, Amp^{r}	[4]
pMo130	Suicide plasmid, *xylE*$^{+}$, *sacB*$^{+}$, Km^{r}	[5]

pIL105	pMo130 plasmid containing gentamicin-resistance cassette, Gm^r	This study
pIL147	pMoΔ*A1S_1772-1773*::Gm^r containing 0.5 kb UP (*A1S_1772*) and 0.5 kb DOWN (*A1S_1773*) fragments; Gm^r	This study
pIL148	pMoΔ*A1S_1799-1800*::Gm^r containing 0.5 kb UP (*A1S_1799*) and 0.5 kb DOWN (*A1S_1800*) fragments; Gm^r	This study

Gm^r, Tp^r, Amp^r genes encoding resistance to gentamicin, trimethoprim, and ampicillin respectively**.**

Table S2. Primers used in this study.

Primer Name	Sequence 5’ → 3’
Ab (17978) glmS FWD	TTCGCTGATGAAAATAGTGG
Ab (17978) glmS REV	ATTCACCTCAAACCGTACAACG
PTn7R	CACAGCATAACTGGACTGATTTC
A1S_1799 Up SphI FWD	atatGCATGCACCTTACAGCCTCTGCAATAC
A1S_1799 NsiI Int REV	atatATGCATGCTGCAAAAATTGCACCTGC
A1S_1800 BamHI Int FWD	atatGGATCCCTTCTCGCCAAATTTCAGC
A1S_1800 Down NotI REV	CGGAGCGGCCGCCTTATATTGTCATTATGGTAC
A1S_1799 Ext FWD	CAGACGTTGTGGTCCCAC
A1S_1801 Ext REV	GTTCCTAAATGGCAGTTATTAGGCG
A1S_1772 Up SphI FWD	CGGAGCATGCATGACCGTATCACCGCTAAAG
A1S_1772 NsiI Int REV	CGGAATGCATCATATTTATGGATCGGGGGAAG
A1S_1773 BamHI Int FWD	atatGGATCCCAAGTTATTTTCTTTCGGCTTTG
A1S_1773 Down NotI REV	CGGAGCGGCCGCCTACTTAATTAATACAAATG
A1S_1772 Ext FWD	ATAGCCTTTAGAAATGAGAAG
A1S_1773 Ext REV	CTCTATAACTATTTAGCAGTTAC
AmfAB_NcoI_FWD	CGCTCCATGGATAACGTAGCTCAGCTAGA
AmfAB_HindIII_REV	CGCAAGCTTCTAGTGGTGGTGGTGGTGGTGCATATTCATTTTCTT
AmfCD_NcoI_FWD	CCTTCCATGGCAAATATACGACCACTCATCGGTTTG
AmfCD_EcoRI_REV	CTTGAATTCTTAGTGGTGGTGGTGGTGGTGTTTGGTCGTATCCACA GAAAC

Figure S1. Expression of AmfB and AmfC proteins in *A. baumannii* IL186 and IL187 cells. Proteins were purified from the isolated membrane fractions, resolved on 12% SDS-PAGE and visualized by immunoblotting with monoclonal anti-6His antibody (Sigma).

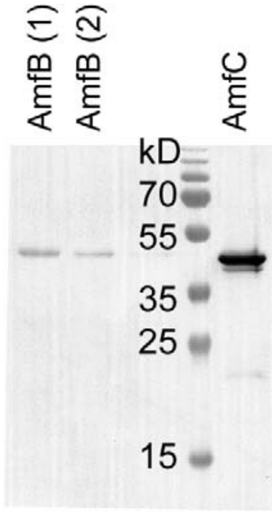

Figure S2. Growth curves of AbWT, Δ2 and AbWT overproducing AmfAB and AmfCD pumps under indicated growth conditions. Growth of Δ2 cells is shown for comparison. Error bars are SD (n=3-6).

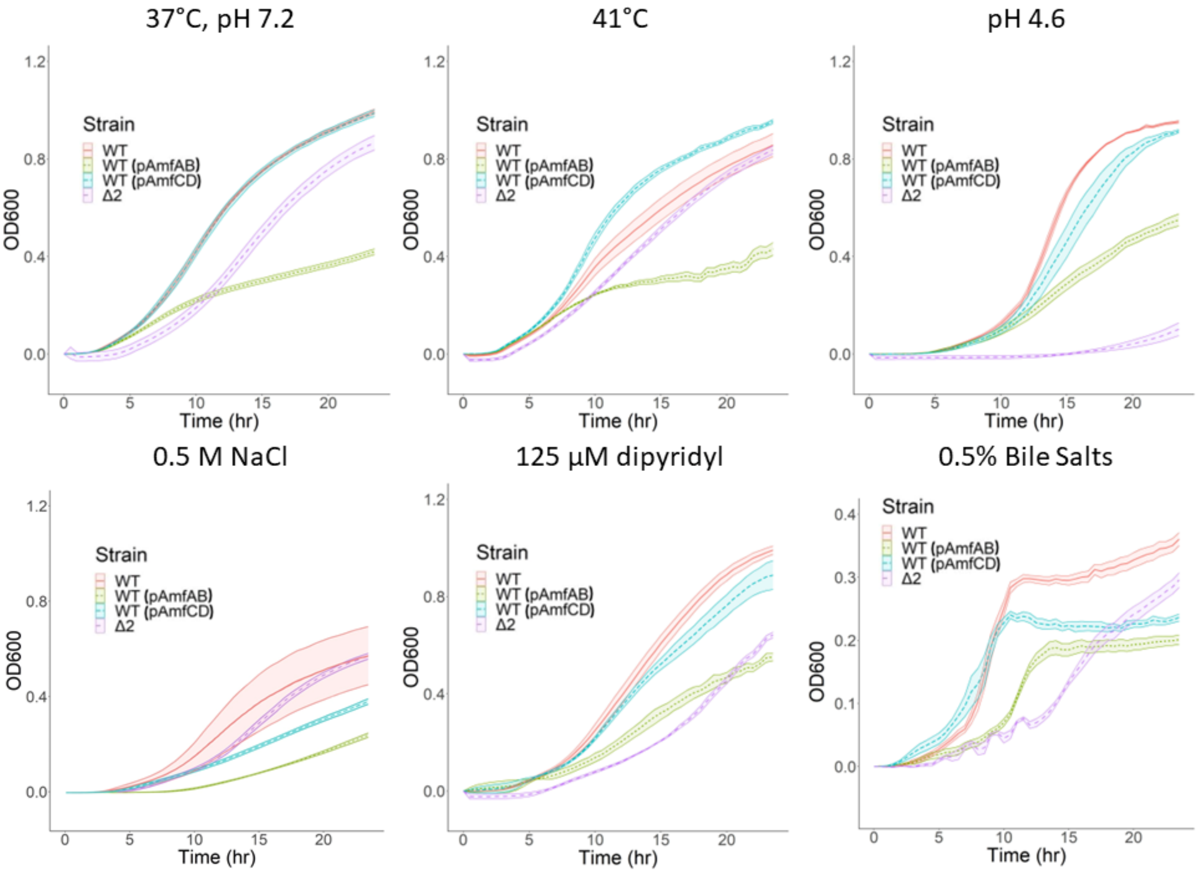

Figure S3. Growth curves of AbWT and its RND-deficient Δ3 cells under indicated growth conditions. Error bars are SD (n=3-6).

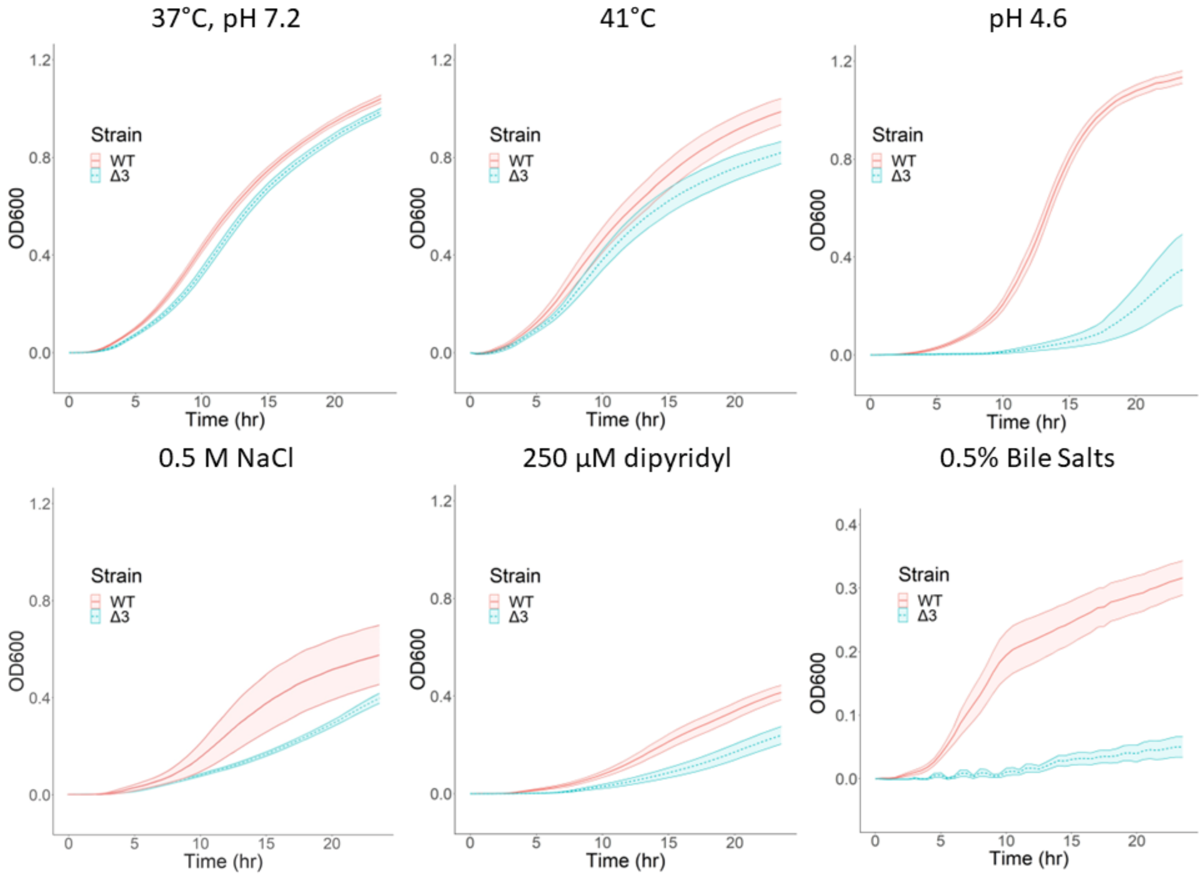

Figure S4. Growth curves of the RND-deficient Δ3 and its derivatives lacking AmfAB, AmfCD or both pumps under indicated growth conditions. Error bars are SD (n=3-6).

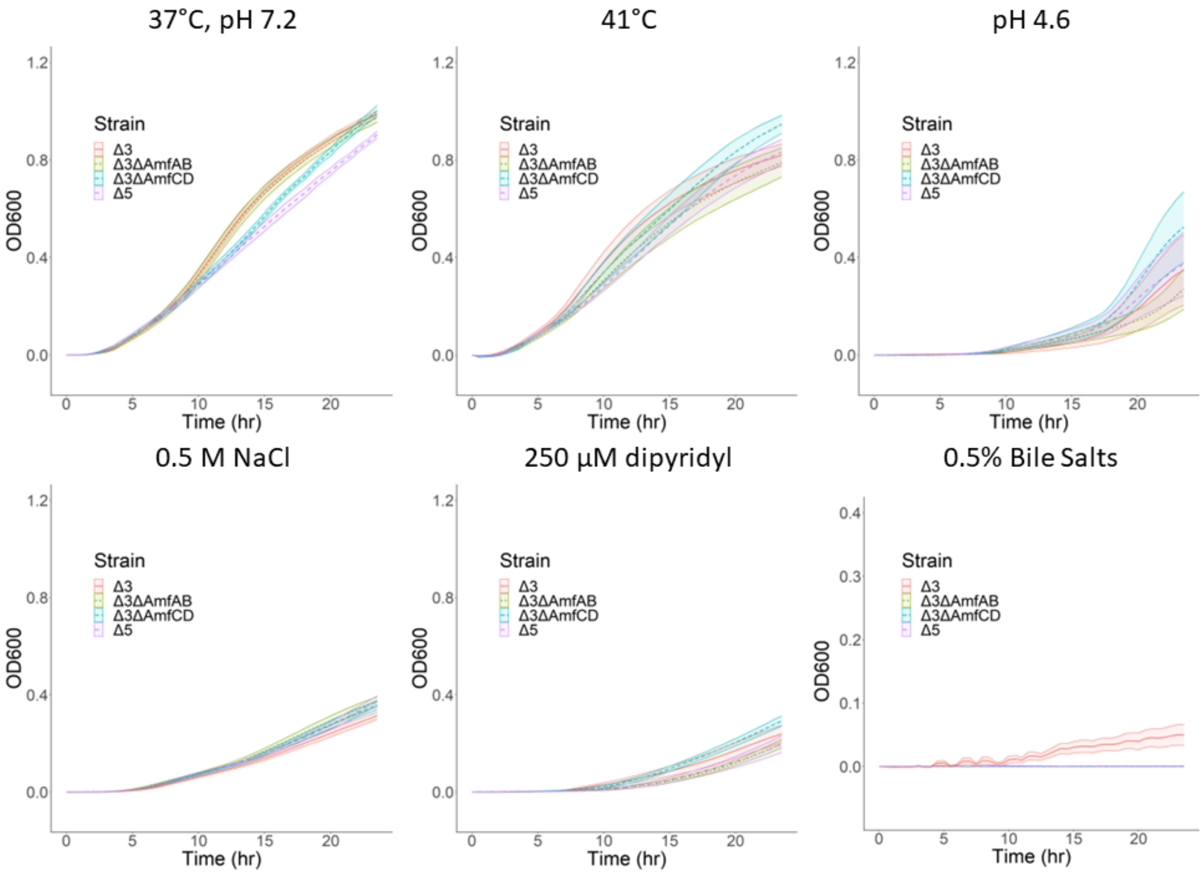

Figure S5. Growth curves of Δ3 overproducing AmfAB and AmfCD pumps under indicated growth conditions. Growth of Δ5 cells is shown for comparison. Error bars are SD (n=3-6).

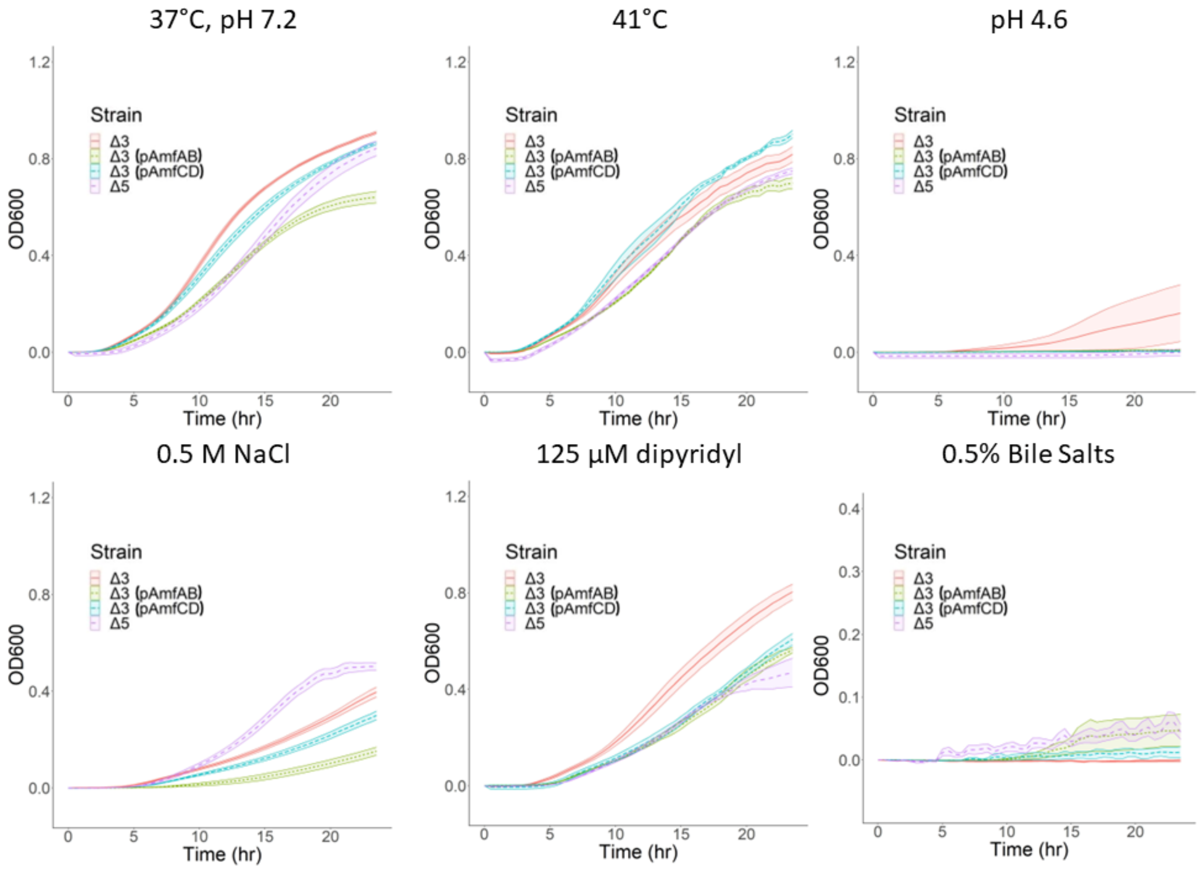

Figure S6. Growth curves of Δ5 overproducing AmfAB and AmfCD pumps under indicated growth conditions. Growth of Δ3 cells is shown for comparison. Error bars are SD (n=3-6).

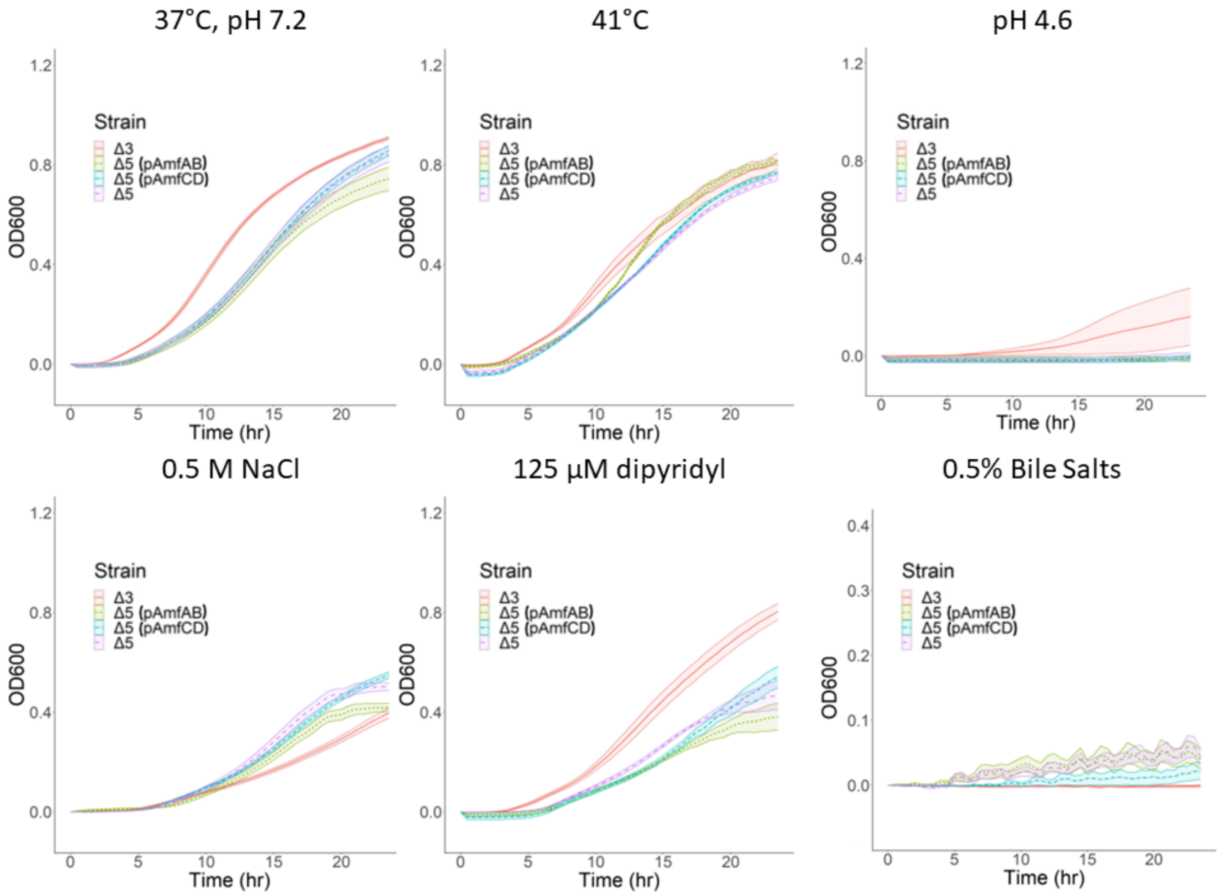

Figure S7. Growth curves of AbWT, the RND-deficient Δ3 and their derivatives lacking both AmfAB and AmfCD pumps. Cells were grown in the M9 based medium supplemented with 0.5% sodium citrate as a sole carbon source and with or without 3.1 μM of $FeCl_3$. Error bars are SD (n=3).

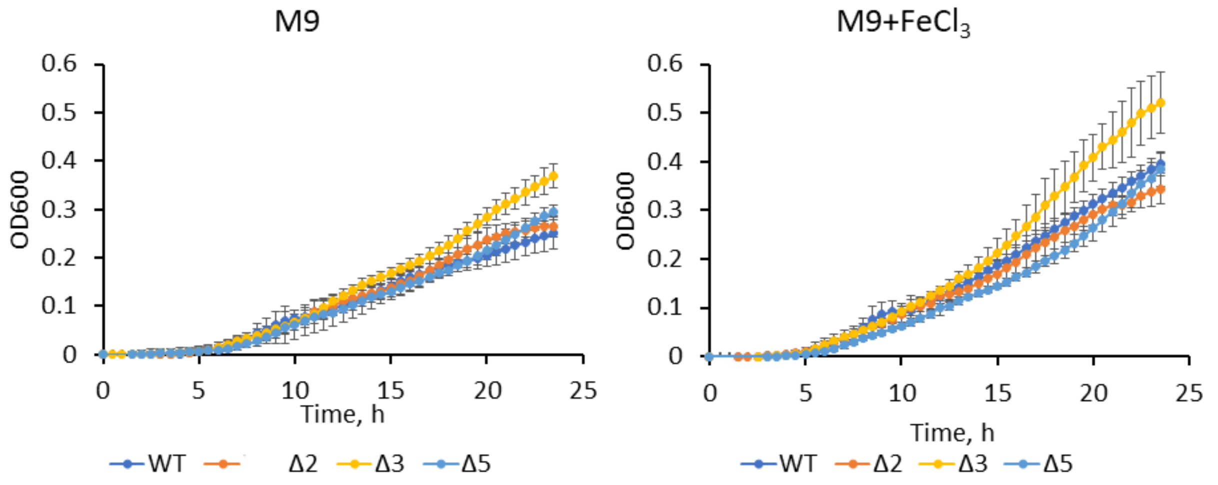

Figure S8. RNAseq analyses of abundances of *amfA* and *amfC* transcripts. A. Measured abundances of transcripts in AB5075 cells stressed by the indicated conditions (data from [6]). B. Changes in the abundances of transcripts in the RND-deficient AB5075 (ΔadeIJK adeB::Tn – ΔBJ) [7] and AB5075 cells stressed by the indicated conditions [6]. Ctrl – LB, 37°C, As – acidic stress, Bs – bile salt stress, Li – low iron, Oss – osmotic stress, and Tm – 41°C.

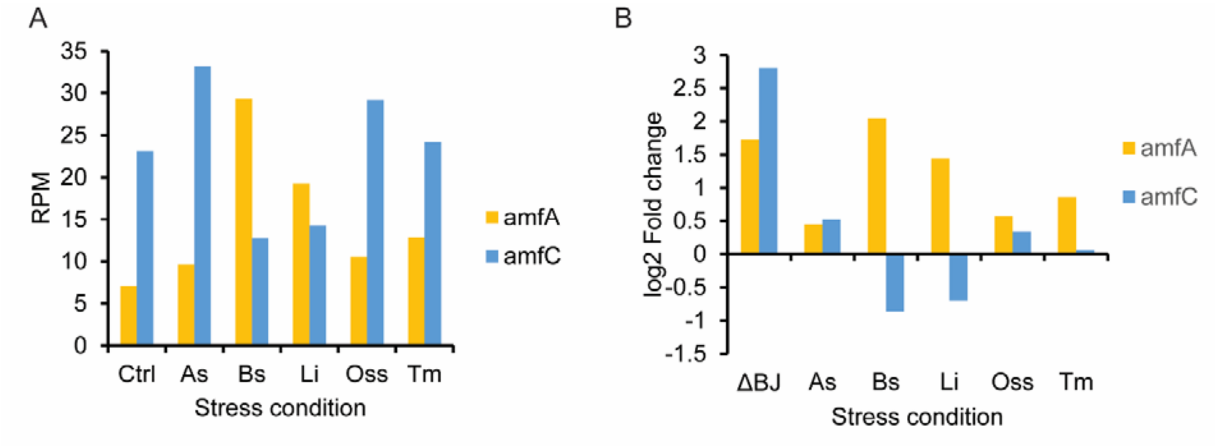

References

1. Krishnamoorthy, G.; Leus, I.V.; Weeks, J.W.; Wolloscheck, D.; Rybenkov, V.V.; Zgurskaya, H.I. Synergy between Active Efflux and Outer Membrane Diffusion Defines Rules of Antibiotic Permeation into Gram-Negative Bacteria. *mBio* **2017**, *8*, 10.1128/mbio.01172-01117, doi:doi:10.1128/mbio.01172-17.
2. Choi, K.H.; Schweizer, H.P. mini-Tn7 insertion in bacteria with single attTn7 sites: example Pseudomonas aeruginosa. *Nat Protoc* **2006**, *1*, 153-161, doi:10.1038/nprot.2006.24.
3. Damron, F.H.; McKenney, E.S.; Barbier, M.; Liechti, G.W.; Schweizer, H.P.; Goldberg, J.B. Construction of Mobilizable Mini-Tn7 Vectors for Bioluminescent Detection of Gram-Negative Bacteria and Single-Copy Promoter lux Reporter Analysis. *Applied and Environmental Microbiology* **2013**, *79*, 4149-4153, doi:10.1128/aem.00640-13.
4. Tucker, A.T.; Nowicki, E.M.; Boll, J.M.; Knauf, G.A.; Burdis, N.C.; Trent, M.S.; Davies, B.W. Defining gene-phenotype relationships in Acinetobacter baumannii through one-step chromosomal gene inactivation. *MBio* **2014**, *5*, e01313-01314, doi:10.1128/mBio.01313-14.
5. Amin, I.M.; Richmond, G.E.; Sen, P.; Koh, T.H.; Piddock, L.J.V.; Chua, K.L. A method for generating marker-less gene deletions in multidrug-resistant Acinetobacter baumannii. *BMC Microbiol* **2013**, *13*, 158-158, doi:10.1186/1471-2180-13-158.
6. Avican, K.; Aldandooh, J.; Togninalli, M.; Mahmud, A.; Tang, J.; Borgwardt, K.M.; Rhen, M.; Fallman, M. RNA atlas of human bacterial pathogens uncovers stress dynamics linked to infection. *NATURE COMMUNICATIONS* **2021**, *12*, doi:10.1038/s41467-021-23588-w.
7. Leus, I.V.; Adamiak, J.; Trinh, A.N.; Smith, R.D.; Smith, L.; Richardson, S.; Ernst, R.K.; Zgurskaya, H.I. Inactivation of AdeABC and AdeIJK efflux pumps elicits specific nonoverlapping transcriptional and phenotypic responses in Acinetobacter baumannii. *Mol Microbiol* **2020**, *144*, 1049-1065, doi:10.1111/mmi.14594.
